# Hot Working Behavior in Multiphase Steel with Ti and V

**DOI:** 10.3390/ma15175852

**Published:** 2022-08-25

**Authors:** Anna Wojtacha, Marek Opiela

**Affiliations:** Department of Engineering Materials and Biomaterials, Silesian University of Technology, 18A Konarskiego Street, 44-100 Gliwice, Poland

**Keywords:** multiphase steel, hot plastic deformation, dynamic recrystallization, thermomechanical simulator

## Abstract

This study investigated the effect of hot working conditions on changes in yield stress and the softening degree in the newly developed multiphase steel with Ti and V microadditions. The research was performed on the GLEEBLE 3800 thermomechanical simulator. In order to determine the σ-ε curves, continuous compression tests were carried out. The samples were plastically deformed at temperatures from 900 °C to 1100 °C at the rate of 0.1 s^−1^, 1 s^−1^ and 10 s^−1^. The activation energy of the plastic deformation was 375 kJ·mol^−1^. The analysis of the shape and course of the curves indicated that the decrease in strain hardening was mainly the result of the continuous dynamic recrystallization process. Two-stage compression with isothermal holding of the samples was also carried out between the two stages of deformation lasting from 1 s to 50 s. The structure of primary austenite was generated using the ARPGE software. The different size of austenite grain is the result of various thermally activated processes—when increasing the strain rate from 0.1 s^−1^ to 10 s^−1^, the average grain size of the primary austenite decreases from approx. 16 µm to approx. 6 µm. The time t_0.5_ needed to form 50% of the austenite fraction recrystallized at 1100 °C is approx. 4 s and extends to approx. 10 s with the reduction in the plastic deformation temperature to 900 °C. The time of complete austenite recrystallization t_R_, which varies from approx. 50 s to approx. 90 s in the tested temperature range, lengthens even more. The obtained results make it possible to develop thermomechanical treatment technology for the production of forgings from the tested multiphase steel.

## 1. Introduction

Modern multiphase steels with retained austenite are the answer to the current requirements of the automotive industry. The requirements for innovative construction materials used for forgings ensure high strength, ductility, fracture resistance (also at reduced temperature) and high fatigue strength [1,2,3,4,5]. Obtaining the expected properties should take place at the lowest possible cost, which is related to the limitation of the amount of alloy additions introduced into the steel [6,7,8,9].

The presence of metastable austenite in the structure, as the phase with the highest plasticity among all structural components, allows for a simultaneous increase in strength and ductility [10,11,12]. The silicon in multiphase steels is added to, preventing the precipitation of cementite in the bainite ferrite and favorably affecting the impact strength of the steel [13,14]. As a result, the retained austenite is enriched with carbon, which increases its share and stabilizes it at room temperature. Multiphase steels have been the subject of research for several years [15,16,17,18,19,20,21,22]; however, the optimal conditions for hot forming and temperature–time profiles of multi-stage cooling have not been developed so far. The major problem is the inhomogeneity retained in austenite grains, in particular the uncontrolled martensitic transformation, which may be the cause of crack initiation and propagation [23,24,25,26,27,28].

The knowledge of the recrystallization process and the size of the grains formed in the previously plastically deformed metal is of significant importance for the design of industrial technologies for hot forming. This ensures that products are obtained not only with the correct geometric form, dimensional tolerances and surface smoothness, but also with the required mechanical properties. In the breaks between stages of deformation, the decrease in strain hardening is a result of static recovery and static recrystallization [29]. The static recrystallization of austenite ends when the growing grains meet and the plastically deformed matrix is exhausted. Then, the recrystallized austenite has a fine-grained structure with a grain size that depends on the chemical composition, the original grain size of this phase before deformation and the conditions of plastic deformation. The interaction between alloying elements and second-phase particles has a great effect on the recovery rate and the mobility of grain boundaries [30]. The influence of dissolved elements (such as Mo, Nb, Ti, and V) comes down to extension of recovery time and reduction in recrystallization rate due to the segregation of atoms of these elements in dislocations lines and on the recrystallization fronts, reducing their mobility [31].

Understanding the course of the thermally activated processes during plastic deformation of the newly developed multiphase steel is of great importance for the development of optimal conditions for the manufacturing technology of forgings. Control of plastic deformation parameters, such as temperature, strain, strain rate and time of breaks between stages of deformation, is aimed at producing metallurgical products with the desired microstructure, stability in terms of strength properties and resistance to cracking under given operating conditions. The purpose of the work was to determine the σ-ε curves and the recrystallization kinetic curves of plastically deformed austenite, and to investigate the microstructure of the newly developed multiphase steel with Ti and V microadditions.

## 2. Material and Methodology

The subject of the research was the newly developed multiphase steel. The chemical composition is listed in Table 1. The chemical composition allows for the production of forgings with a multiphase structure with retained austenite. About 1% of silicon was introduced into the steel, which during the bainite transformation will inhibit the precipitation of carbides, and the excess carbon will diffuse into the austenite, favoring its thermal stabilization [32]. The Mo addition and the Ti and V microadditions introduced into the steel are designed to refine and strengthen the structure. Ti and V form TiN, TiC and VC that during austenitization inhibit the growth of the austenite grains.

The metallic charge of Armco iron with the addition of synthetic graphite, FeSi75A ferroalloy and a specified amount of pure Mo, Ti and V, was melted in an electric induction furnace VEM I20 (VEM, California City, CA, USA). In the steel melting process, silica sand was used as a slag-forming material. The pouring temperature was measured with a Pt-PtRh10 thermelement (Matmatch GmbH, München, Germany). The temperature was 1650 °C. The chemical composition was determined using a LECO GDS500A glow discharge emission spectrometer (Leco Inc., St. Joseph, MO, USA).

**Figure 1:**
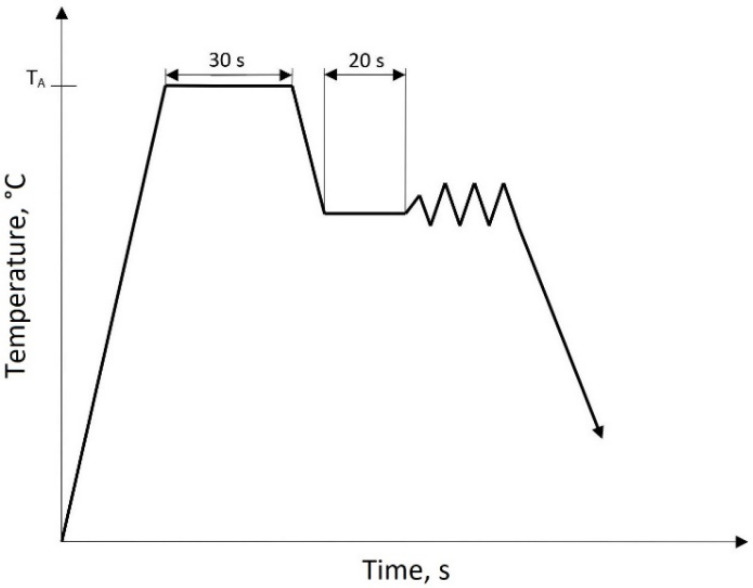
Diagram of continuous compression of samples.

The plastic deformation process using the GLEEBLE 3800 thermomechanical simulator (Dynamic Systems Inc., Poestenkill, NY, USA) was carried out on axisymmetric samples with a diameter of 10 mm and a length of 15 mm. In order to determine the σ-ε curves and the activation energy, continuous compression tests of the samples to the deformation φ = 0.69 were carried out. Graphite–tantalum foils were used to reduce friction between the faces of the samples and the anvil surface. The samples were heated in a vacuum at a rate of 3 °C/s to the austenitizing temperature of 1150 °C, annealed for 30 s and cooled to the following plastic deformation temperatures: 900 °C, 950 °C, 1000 °C, 1050 °C and 1100 °C. This is the typical temperature range used in the thermomechanical treatment of microalloyed steels. The diagram of the continuous compression of the samples is presented in Figure 1. The compression of the samples was performed at the rate of 0.1 s^−1^, 1 s^−1^ and 10 s^−1^. After the desired deformation, the samples were quenched in water.

The activation energy of the hot plastic deformation process was determined using the Energy 4.0 computer program (VSB, Technical University of Ostrava, Ostrava, Czech Republic) based on the equation [33]:

(1) $$\dot{\varepsilon }=A{\left[\sin(\alpha \sigma )\right]}^{n}\cdot \exp(-\frac{Q}{\mathrm{RT}})$$

where:

A, α, n—constants.
T—Deformation temperature.
$\dot{\varepsilon }$—Strain rate.
σ—Stress value corresponding to the maximum yield stress.
R—Gas constant = 8.314 J·mol^−1^·K^−1^.

In order to designate the kinetics of plastically deformed austenite recrystallization, intermittent compression tests were carried out at the rate of 10 s^−1^ for a given actual strain of 0.2. The tests were carried out at temperatures from 900 °C to 1100 °C with the isothermal holding between two stages of deformation. The isothermal holding lasted from 1 s to 50 s. The degree of softening (the decrease in strain hardening) was determined using the following relationship [33]:

X = (σ_1_ − σ_2_)/(σ_1_ − σ_0_) (2)

where:

σ_0_—stress needed to begin plastic deformation.
σ_1_—stress that occurred during the interrupted plastic deformation in the first stage.
σ_2_—stress required to begin plastic deformation in the second step after Δt between these steps.

The kinetics of plastically deformed austenite recrystallization was featured using the Johnson–Mehl equation [33]:

y = 1 − exp(−k·t^n^) (3)

where:

y—Part of recrystallized austenite after time t.
k—Constant.
n—Exponent.

In order to carry out research with the light microscope, metallographic specimens were prepared. The sections were made in the plane consistent with the sample axis at a distance of 1/3 from the center of the sample, and then ground on SiC-based paper and polished. Etching with 5% Nital was used to reveal the structure of the steel. Metallographic observations were made with the Zeiss Observer.Z1m light microscope (Carl Zeiss AG, Oberkochen, Germany). Examination of the microstructure using the Electron Backscatter Diffraction (EBSD) method was carried out with the SUPRA 25 scanning electron microscope by Zeiss (Carl Zeiss AG, Oberkochen, Germany). In order to reveal the boundaries of the primary austenite grains, the ARPGE software (Cyril Cayron, Lausanne, Switzerland) was used, which enabled automatic reconstruction of the primary austenite structure based on the EBSD analysis [34].

## 3. Results

### 3.1. Plastometric Test Results

**Figure 2:**
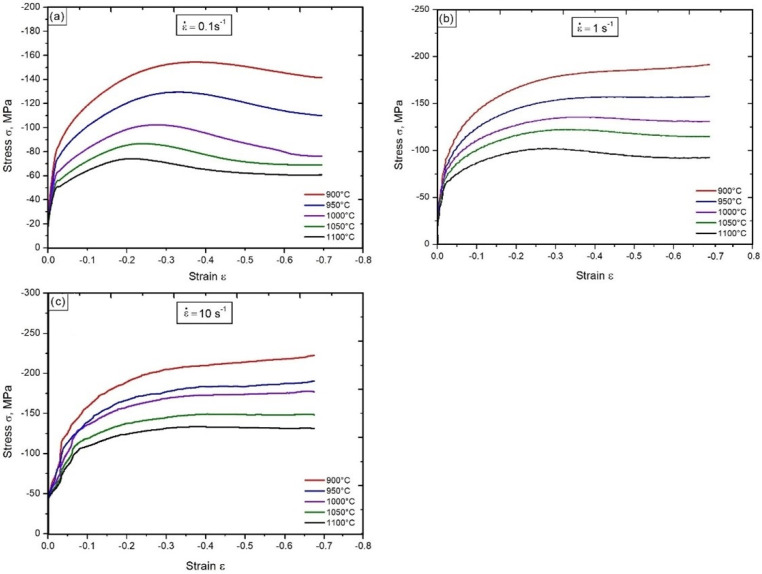
Impact of temperature and strain rate on the course of the σ–ε curves: 0.1 s^−1^ (**a**), 1 s^−1^ (**b**), 10 s^−1^ (**c**).

The conducted continuous compression tests with the use of the GLEEBLE 3800 thermomechanical simulator permitted us to determine the influence of deformation parameters on the strengthening curves σ–ε and the deformation ε_m_ corresponding to the maximum value of the yield stress. This allowed us to estimate the deformation necessary to initiate dynamic recrystallization of austenite (ε_cd_ ≈ 0.8ε_m_) under the tested conditions. The tests were carried out at temperatures from 900 °C to 1100 °C with the rate of 0.1 s^−1^, 1 s^−1^ and 10 s^−1^ (Figure 2).

In the first phase of compression with strain rate 0.1 s^−1^, within the strain hardening range ε < 0.025, a significant increase in yield stresses occurred on the σ–ε curves, which was caused by the increasing density of dislocations created in this process. When the strain rate increased to the values of 1 s^−1^ and 10 s^−1^, the yield stress increased, and the strain hardening range increased to ε < 0.028 and ε < 0.035, respectively. Instabilities, especially in the initial phase in Figure 2c, were due to the way the experiment was carried out. In the next phase of compression, when the strain increased to ε = ε_m_, the stress increase was milder. This was the effect of simultaneous generation by sources of new dislocations during plastic deformation and the course of thermally activated processes, causing a fragmentary disappearance of the emitted dislocations. For the deformation from ε = ε_m_ to ε = 0.69, the strengthening curves were characterized by a slight decrease in yield stresses until they reached the equilibrium state between the strengthening processes and its decrease due to the course of thermally activated processes.

The shape and course of the curves gained in the compression with the rate of 0.1 s^−1^ (Figure 2a) indicate that the decrease in strain hardening in the applied temperature range was the effect of a continuous dynamic recrystallization process. Curves of the tested steel obtained after compression with the rate of 1 s^−1^ are presented in Figure 2b. In this case, dynamic recrystallization is a process that controls the course of plastic deformation at temperatures from 1000 °C to 1100 °C. At 900 °C and 950 °C, this process was dynamic recovery. During compression at the rate of 10 s^−1^, the decrease in strain hardening in the temperature range of 1050 °C to 1100 °C was the result of the dynamic recrystallization process, while at temperatures of 900 °C, 950 °C and 1000 °C, dynamic recovery was the dominant process. The conducted tests correspond to the results of calculating the activation energy of the plastic deformation. The activation energy of the plastic deformation process was Q = 375 kJ·mol^−1^, while the constant values in Equation (1) for the stresses corresponding to the deformations ε_m_ were: A = 3.02 × 1012, α = 0.016 and n = 3.9. The values of Q, α and n were determined by the Energy 4.0 computer program. The obtained value of activation energy of the plastic deformation was higher than the self-diffusion activation energy, when the process controlling the plastic deformation was dislocation climbing and sub-grain formation. The self-diffusion activation energy for the tested steel was 290 kJ·mol^−1^. Similar values of activation energy of the plastic deformation for microalloyed steels were obtained in the works [35,36] for steels with a similar chemical composition.

**Figure 3:**
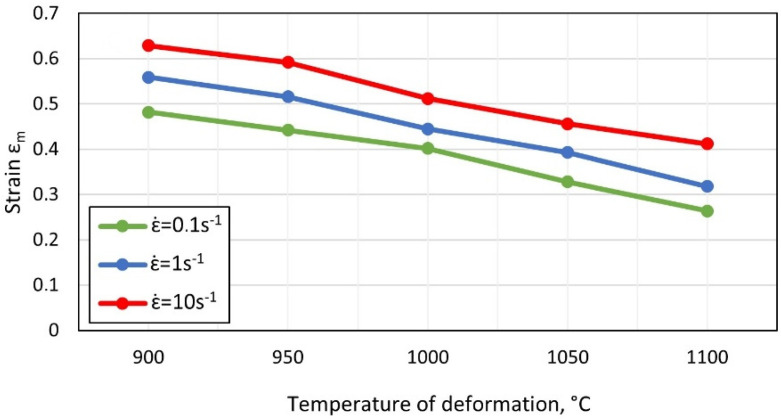
Impact of temperature and strain rate on the value of deformation ε_m_.

The impact of the temperature and the strain rate on the deformation ε_m_, suitable for the maximum values of yield stress, are shown in Figure 3.

Along with lowering the temperature and increasing the strain rate, an increase in the value of strain ε_m_ was observed. For a strain rate of 0.1 s^−1^, the reduction in the plastic deformation temperature from 1100 °C to 900 °C caused an increase in the maximum yield stress σ_m_ from approx. 78 MPa to 160 MPa, and deformation ε_m_ from 0.26 to 0.48. The reduction in the deformation temperature of the compression temperature at the rate of 1 s^−1^ influenced the increase in σ_m_ from 117 MPa to 194 MPa. For the parameters described, there was a simultaneous increase in ε_m_ from 0.32 to 0.56. During the compression of steel with the rate of 10 s^−1^ at 900 °C, the value of σ_m_ was 221 MPa, with ε_m_ equal to 0.63. With the increase in the deformation temperature to 1100 °C, the value of σ_m_ decreased to 143 MPa, with ε_m_ equal to 0.41.

**Figure 4:**
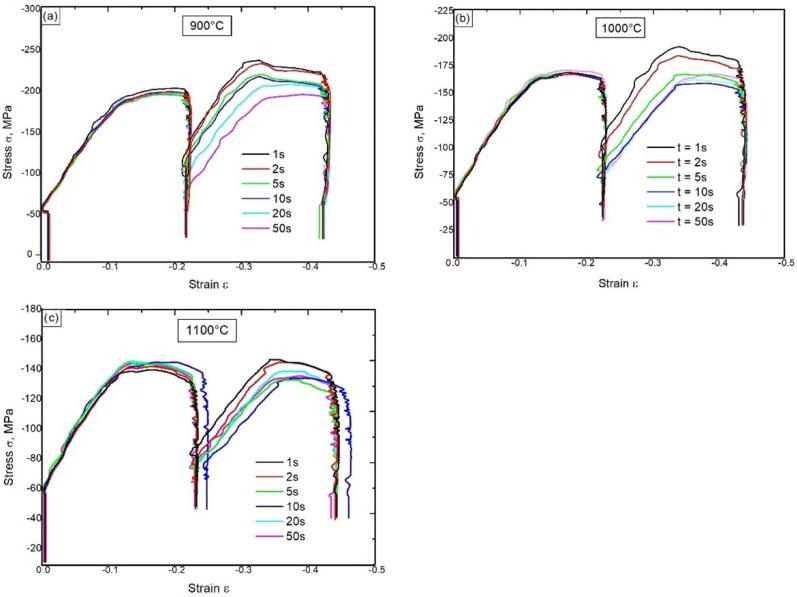
The σ–ε curves obtained during the two-stage compression at the rate of 10 s^−1^ with the use of intervals between successive stages of deformation of 1 s, 2 s, 5 s, 10 s, 20 s and 50 s; the temperature: 900 °C (**a**), 1000 °C (**b**), 1100 °C (**c**).

Then, two-stage hot compression tests were carried out, which allowed us to determine the influence of the test temperature on the kinetics of thermally activated processes. As expected, the intermittent compression tests for a given deformation showed that during the isothermal holding between the deformation stages, the strain hardening was partially lost. The course of the decrease in the strain hardening process depended on the isothermal holding time and the strain temperature. It was a result of the processes of static recovery and static recrystallization. The obtained σ-ε curves recorded during compression with a rate of 10 s^−1^ at 900 °C, 1000 °C and 1100 °C using the isothermal holding of the samples for 1 s, 2 s, 5 s, 10 s, 20 s and 50 s between the first and second deformation are shown in Figure 4. The degree of the strain hardening reduction between stages of deformation depends on the values of σ_2_ and σ_0_ occurring in Equation (2).

**Figure 5:**
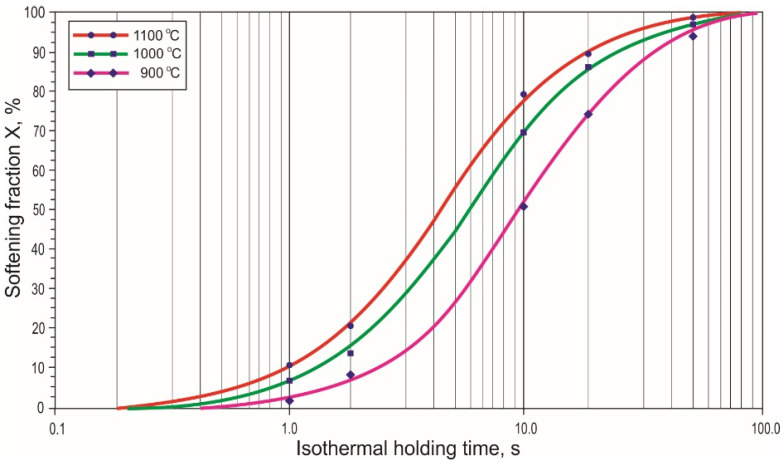
Influence of temperature on the kinetics of static recrystallization in the hot compression test; strain rate 10 s^−1^.

The influence of the microalloying elements dissolved in the solid solution and the dispersive particles of the MX phases on the recovery rate and mobility of the recrystallization fronts had a significant impact on the kinetics of the static recrystallization of the tested steel. This interactivity is presented by the curves of the decrease in strain hardening of austenite (Figure 5). The collection of the curves in this figure shows that lowering the temperature of plastic deformation and isothermal annealing causes a significant prolongation of the recovery time and a decrease in the rate of austenite recrystallization. This is an effect of the decreasing value of the iron self-diffusion ratio and the volumetric diffusion ratios of the alloy components, along with the lowering of the temperature and the impact of their atoms and dispersive particles of interstitial phases introduced into the steel of Ti and V microadditions on the migration rate of the recrystallization fronts. At 1100 °C additions V, Mo and partially Ti were dissolved in austenite [37].

The effect of dissolved elements consists of extending the recovery time and reducing the recrystallization rate due to the segregation of element atoms in the dislocation deformation field and on the recrystallization fronts [38]. The greatest impact on the extension of recovery and recrystallization times was exerted by the interaction of the segregation of dissolved atoms and dispersion particles of interstitial phases. As can be seen from the data presented in Figure 5, the time t_0.5_ needed to form 50% of the austenite fraction recrystallized at 1100 °C was approx. 4 s and extended to approx. 10 s as the plastic deformation temperature reduced to 900 °C. The time of entire recrystallization of austenite t_R_ increased even more, varying from approx. 50 s to approx. 90 s in the tested temperature range.

The course of the kinetic curves indicates the eventuality of their description in compatibility with the model of recrystallization after hot deformation, taking into account the process of static recovery, static and metadynamic recrystallization. At the lower temperature, the applied deformation degree was lower than that required to initiate the dynamic recrystallization process. After deformation at 900 °C with a rate of 10 s^−1^, the recovery and static recrystallization was responsible for the decrease in strain hardening, and at 1000 °C and 1100 °C, static recovery, static recrystallization and metadynamic recrystallization.

### 3.2. Microstructural Evaluations

**Figure 6:**
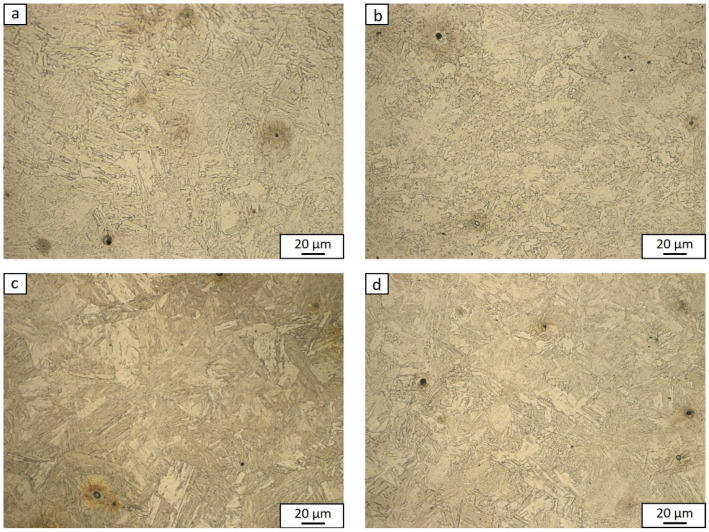
Martensitic structure with a small part of retained austenite of steel hardened in water after deformation: 0.2 (**a**,**c**) and 0.69 (**b**,**d**); plastic deformation temperature: 900 °C (**a**,**b**); 1100 °C (**c**,**d**).

After plastometric tests, metallographic tests were carried out, which allowed us to determine the impact of plastic deformation parameters on the structure of the analyzed steel. For example, Figure 6 shows the structures of the test steel revealed after the continuous compression of the samples at a temperature of 1100 °C with the rate of 1 s^−1^. The samples of the tested steel, hardened in water after deformation with a value of 0.2 and 0.69, showed a martensitic structure with a slight indication of retained austenite, the presence of which was confirmed by the authors in previous works [39,40]. As expected, the deformed samples with a higher degree of deformation showed greater fragmentation of the structure (Figure 6b,d).

**Figure 7:**
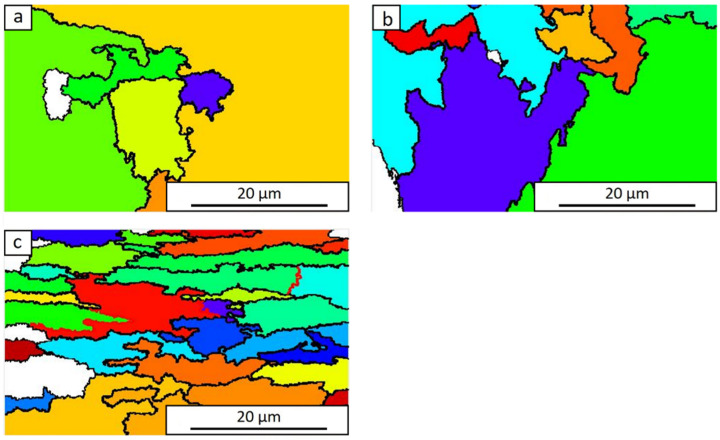
Primary austenite grain boundaries generated by the ARPGE program after continuous compression of samples at 1100 °C for deformation: 0.2 (**a**), 0.4 (**b**), 0.69 (**c**).

The results of the microstructure generated by the ARPGE software (primary austenite grains of the tested steel) after the continuous compression tests at a temperature of 1100 ° C with a variable degree of deformation are presented in Figure 7. The exact values of the grain size measurements with changing values of deformation are presented in Table 2.

**Figure 8:**
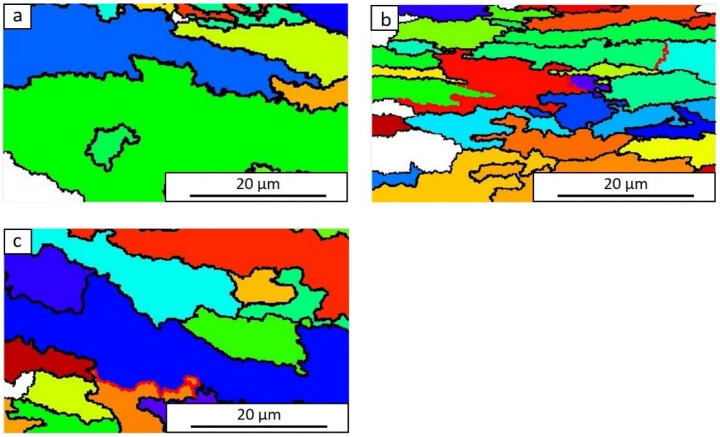
Primary austenite grain boundaries generated by the ARPGE program after continuous compression of samples at 1100 °C for deformation 0.69, at the rate: 0.1 s^−1^ (**a**), 1 s^−1^ (**b**), 10 s^−1^ (**c**).

The results of microscopy observations of the primary austenite grains generated by the ARPGE program after continuous compression of the samples at 1100 °C with a variable strain rate are shown in Figure 8. The exact values generated for the grain size measurements at changing strain rates are shown in Table 3. Recrystallization takes place at individual stages of continuous compression; the different size of austenite grain was the effect of the course of various thermally activated processes. When increasing the strain rate from 0.1 s^−1^ to 1 s^−1^, the average grain size of the primary austenite decreased from approx. 16 µm to approx. 8 µm. Increasing the rate of deformation caused an increase in the yield stress, which initially increased with decreasing intensity to the maximum value, and then decreased to a certain predetermined value [41]. At the strain rate of 10 s^−1^, there was a reduction in the average grain size of the primary austenite to about 6 µm.

## 4. Discussion

Tests of the hot plastic deformation process carried out at temperatures from 900 °C to 1100 °C with the use of deformation rates of 0.1 s^−1^, 1 s^−1^ and 10 s^−1^ allowed us to determine the influence of compression parameters on the course of the strengthening curves (Figure 2). Tests of continuous compression of samples on the GLEEBLE 3800 simulator showed that with a decrease in temperature and an increase in the rate of deformation, the deformation value ε_m_, suitable for the maximum value of the yield stress moves towards greater deformation. This relationship has been confirmed by Liu et al. [42], as well as in the works [43,44,45]. The analysis of the shape of the curves shows that in the tested range of hot deformation parameters, the decrease in deformation hardening of the steel significantly depends on the temperature and the rate of deformation. For a strain rate of 0.1 s^−1^ in the entire temperature range, the process controlling the course of plastic deformation is dynamic recrystallization. During the compression of the samples at the rate of 1 s^−1^, the decrease in strain hardening is the result of dynamic recrystallization in the temperature range from 1000 °C to 1100 °C and dynamic recovery in the temperature range from 900 °C to 950 °C. During the compression of samples at the rate of 10 s^−1^, the process responsible for the decrease in strain hardening at 1050 °C and 1100 °C is dynamic recrystallization, and at lower temperatures, dynamic recovery. Similar results were achieved in the articles [46,47,48]. The activation energy of the plastic deformation process is 375 kJ·mol^−1^. Similar values of the activation energy of the plastic deformation process—for steel with microadditions with a chemical composition similar to the analyzed steel—were obtained in the works [35,36].

Similar issues concerning the influence of hot working conditions on the change in yield stress and hardening were the subject of research in the works [37,49,50,51,52]. For example, in [37], investigations were carried out to determine the conditions of hot working of two microalloyed steels, type Ti-V (steel A) and Ti-Nb-V (steel B). The tested steels with similar C content were characterized by different content of microadditions. Steel A contained 0.033% Ti and 0.008% V, while steel B—0.028% Ti, 0.027% Nb and 0.019% V. Continuous compression tests of the samples were carried out on the Gleeble 3800 thermomechanical simulator, and consisted of austenitizing the samples at a temperature of 1150 °C for 30 s, cooling to the desired deformation temperature (in the range from 900 °C to 1100 °C) and subsequently cooling to the ambient temperature. The samples were compressed at a rate of 1 s^−1^, 10 s^−1^ and 50 s^−1^. The values of the maximum yield stresses for steel A compressed at the rate of 1 s^−1^ varied in the range from 98 MPa to 186 MPa, respectively for the temperature in the range from 1100 °C to 900 °C. For the strain rate of 50 s^−1^, the values of the maximum yield stress varied from 169 MPa to 285 MPa in the same temperature range. In the case of steel with microaddition Nb (steel B) at the deformation rate of 1 s^−1^, the maximum yield stresses values changed from 103 MPa to 192 MPa, respectively for the temperature in the range from 1100 °C to 900 °C. For the strain rate of 50 s^−1^, the values of the maximum yield stresses varied in the range from 173 MPa to 285 MPa in the same temperature range. The conducted experiment showed that the process controlling the course of plastic deformation in the case of these steels is dynamic recrystallization with a continuous course. The determined activation energy of the plastic deformation process was 382 kJ·mol^−1^ for Ti-V steel and 398 kJ·mol^−1^ for Ti-Nb-V steel. These values are slightly higher than the determined activation energy value for the analyzed steel, which is the result of a different chemical composition [37].

Nucleation and an increase in recrystallization significantly affect the formation of austenite, its spatial distribution and the resulting microstructure morphology [49,50]. Applying recrystallization kinetics can be seen as a way of controlling the final microstructure and mechanical properties by adding some of the recrystallization delaying micro-additions. In the works [51,52], the authors demonstrated the effectiveness of the influence of Ti and Nb microadditions in inhibiting the recrystallization process. Bellavoine et al. [52] showed that Ti and Nb are released as carbonitrides of the (Ti, Nb) (C, N) type with possible synergy between Ti and Nb, and proposed modeling methods rationalizing the effect of microadditions on recrystallization. The tested steel is characterized by a fine-grained structure resulting from the presence of precipitates containing Ti and V microadditions, which inhibit the growth of austenite grains. Moreover, it was found that in steels with a coarse-grained structure of austenite, there is a smaller number of potential sites for dynamic recrystallization nucleations [52].

Conducted intermittent compression tests of samples to a given deformation with the use of isothermal holding for a time from 1 s to 50 s allowed us to determine the kinetic curves of austenite recrystallization (Figure 5). The chemical composition of the steel, especially the interaction of Mo, Cr and Ti microadditions, determined that the thermal activated processes causing the decrease in strain hardening of austenite after plastic deformation are relatively slow, so the γ phase recrystallization time is long, especially at 900 °C, which is often the end of the plastic deformation temperature. The time of complete recrystallization of austenite t_R_ at temperatures from 1100 °C to 900 °C varies from approx. 50 s to 90 s. Moreover, there is significant deformation at high rate and short breaks for the transfer of the produced element from one die pattern to another. They do not create favorable conditions for the course of static recrystallization, which would enable the grinding of austenite grains [53]. Therefore, for technical purposes—especially in the case of microalloyed steels—the time t_0.5_ is more important than the t_R_ time, which allows for the production of 50% of the recrystallized austenite fraction. Obtaining this proportion of statically recrystallized austenite requires an ability to withstand approx. 4 s at 1100 °C and approx. 10 s at 900 °C.

## 5. Conclusions

Based on the research, the succeeding conclusions can be drawn:

- The analysis of the shape and course of the curves received in the continuous compression test shows that the decrease in strain hardening in the applied temperature range is mainly the result of the continuous dynamic recrystallization process. This is also confirmed by calculations of the activation energy of the process of plastic deformation of the tested material.
- Due to the different degree of recrystallization taking place at the individual stages of continuous compression, the different size of austenite grain is the effect of various thermally activated processes. When increasing the strain rate from 0.1 s^−1^ to 10 s^−1^, the average grain size of the primary austenite decreases from approx. 16 µm to approx. 6 µm.
- The time t_0.5_ needed to form 50% of the austenite fraction recrystallized at 1100 °C is approx. 4 s and extends to approx. 10 s with the reduction in the plastic deformation temperature to 900 °C. The time of complete austenite recrystallization t_R_, which varies from approx. 50 s to approx. 90 s in the tested temperature range, lengthens even more.
- The obtained results make it possible to develop thermomechanical treatment technology for the production of forgings from the tested multiphase steel.

## Figures and Tables

**Table 1:** Chemical composition of the tested steel [wt%].

C	Si	Mn	Cr	Mo	Ti	V	P	S
0.175	1.020	1.870	0.028	0.218	0.031	0.022	0.014	0.020

**Table 2:** Results of grain size measurements as a function of deformation value; deformation rate: 1 s^−1^.

Deformation Value	Smallest Grain Size [µm]	Largest Grain Size [µm]	Average Grain Size [µm]
0.2	7.14	27.04	20.16
0.4	3.3	38.1	18.7
0.69	3.0	12.5	8.2

**Table 3:** Results of grain size measurements as a function of strain rate; deformation value: 0.69.

Rate of Deformation [s^−1^]	Smallest Grain Size [µm]	Largest Grain Size [µm]	Average Grain Size [µm]
0.1	4.6	30.5	16.0
1	3.0	12.5	8.2
10	2.3	7.4	6.1

## Data Availability

Not applicable.
